# MIRIAD—Public release of a multiple time point Alzheimer's MR imaging dataset

**DOI:** 10.1016/j.neuroimage.2012.12.044

**Published:** 2013-04-15

**Authors:** Ian B. Malone, David Cash, Gerard R. Ridgway, David G. MacManus, Sebastien Ourselin, Nick C. Fox, Jonathan M. Schott

**Affiliations:** a Dementia Research Centre, UCL Institute of Neurology, Queen Square, London, WC1N 3BG, UK; b Centre for Medical Image Computing, UCL, Gower Street, London, WC1E 6BT, UK; c NMR Research Unit, UCL Institute of Neurology, Queen Square, London, WC1N 3BG, UK; d Wellcome Trust Centre for Neuroimaging, UCL Institute of Neurology, Queen Square, London WC1N 3BG, UK

**Keywords:** Alzheimer's, MRI, Longitudinal, Imaging, Database

## Abstract

The Minimal Interval Resonance Imaging in Alzheimer's Disease (MIRIAD) dataset is a series of longitudinal volumetric T1 MRI scans of 46 mild–moderate Alzheimer's subjects and 23 controls. It consists of 708 scans conducted by the same radiographer with the same scanner and sequences at intervals of 2, 6, 14, 26, 38 and 52 weeks, 18 and 24 months from baseline, with accompanying information on gender, age and Mini Mental State Examination (MMSE) scores. Details of the cohort and imaging results have been described in peer-reviewed publications, and the data are here made publicly available as a common resource for researchers to develop, validate and compare techniques, particularly for measurement of longitudinal volume change in serially acquired MR.

## Introduction

Alzheimer's disease (AD) is an increasingly prevalent problem as the population ages. Structural MR imaging is a well established, widely available, non-invasive tool for investigating the inevitable downstream consequence of neurodegeneration in Alzheimer's disease—atrophy. In the correct clinical context the presence of medial temporal lobe atrophy on cross-sectional MR imaging has positive predictive value for a diagnosis of AD and this is now incorporated in diagnostic criteria (Dubois et al., 2007; McKhann et al., 2011). Quantification of the rate of global and regional brain volume loss from serially acquired MRI is increasingly used both to understand the progression of the disease and as an outcome measure for clinical trials, with the premise that a disease-modifying therapy would be expected to slow the rate of atrophy towards that seen in normal ageing (Fox and Schott, 2004). Numerous methods for quantifying change between serial scans have been proposed and are used both in observational and therapeutic studies. In the absence of a “gold standard” validating new methods and comparing the performance of different algorithms are not straightforward.

We announce here the public release of a longitudinal database of structural (T1 contrast) MRI scans from the Minimal Interval Resonance Imaging in Alzheimer's Disease (MIRIAD) study. Whilst there are a number of other open imaging databases, including the Alzheimer's Disease Neuroimaging Initiative (ADNI, Jack et al., 2010), the Australian Imaging, Biomarker & Lifestyle Flagship Study of Ageing (AIBL, Ellis et al., 2010) and Open Access Series of Imaging Studies (OASIS, Marcus et al., 2010) the MIRIAD dataset has a number of features making it ideal for methods development and validation. These include:

1. all scans on the same scanner over the same time period;
2. multiple serial scans (up to nine over two years) for both AD and controls;
3. a scanning schedule designed to give a wide range of inter-scan intervals from 2 weeks to 2 years; and
4. back-to-back scanning at three time points.

These features allow for formal assessments of the symmetry, transitivity and reproducibility of measures of atrophy, and assessment of bias (Fox et al., 2011). By making this dataset widely available, the performance of different image analyses methodologies can be compared directly; and novel tools can be validated before being applied to other datasets.

## Overview

The MIRIAD study was principally motivated to establish the minimal interval over which it would be feasible to undertake clinical trials in Alzheimer's disease using atrophy measured from serial MRI as an outcome measure. Supplementary aims were:

1. To assess whether combining more than two scanning time points would increase statistical power and, if so, the optimal combination and timing of scans for trials of varying lengths.
2. To provide a means of assessing the reproducibility of techniques within a single day and over short intervals where changes in individual's hydration and scanner fluctuations, but not pathological atrophy, might be expected.

Details of subject demographics have been previously described (Schott et al., 2006) and are summarised here in Table 1. In brief the dataset includes 46 patients with a diagnosis of mild–moderate probable AD (NINCDS–ADRDA, McKhann et al., 1984) all seen at the Dementia Research Centre, Institute of Neurology, UCL, and 23 non-demented control subjects, typically the patient's spouse or carer. Inclusion criteria included age over 55 years and a mini-mental state examination (MMSE, Folstein et al., 1975) score between 12 and 26/30. Controls had MMSE scores > 26/30, and no history of cognitive impairment, head injury, major psychiatric disease or stroke. Exclusion criteria included any neurodegenerative disease (apart from AD for the patients), or inability to tolerate MRI. At baseline and one year all subjects underwent a detailed neuropsychometric and clinical evaluation. MMSE score was recorded at baseline and 6-monthly intervals. All subjects were requested to attend seven imaging visits at 0, 2, 6, 14, 26, 38 and 52 weeks from baseline. 39 subjects who completed all these visits during the study attended a further scan at 18 months, 22 of these had a further scan at 24 months. At 0, 6 and 38 weeks two back-to-back scans were conducted. Study attendance figures are shown in Table 2. Ethical approval for the study (and subsequently its release) was received from the local research ethics committee, and written consent obtained from all participants.

All scans were conducted on the same 1.5 T Signa MRI scanner (GE Medical systems, Milwaukee, WI) and acquired by the same radiographer. Three-dimensional T1-weighted images were acquired with an IR-FSPGR (inversion recovery prepared fast spoiled gradient recalled) sequence, field of view 24 cm, 256 × 256 matrix, 124 1.5 mm coronal partitions, TR 15 ms, TE 5.4 ms, flip angle 15°, TI 650 ms. A total of 708 volumetric scans were acquired with up to 12 scans per individual, 25 different interscan intervals, allowing for a total of 2199 scan-pairs for patients with AD and 1182 scan-pairs for controls.

## Results to date

A list of published articles which have used (in full or in part) this dataset is shown in Table 3. Amongst others, these have included a number of methodological papers describing techniques for estimating hippocampal atrophy using a hippocampal template and the boundary shift integral (Barnes et al., 2007a), fluid registration (Barnes et al., 2007b), an automated medial temporal lobe scale (Ridha and Barnes, 2007) and label fusion methods (Leung et al., 2010). Clinical papers have included assessments of hippocampal asymmetry in AD (Barnes et al., 2005); the effect of APOE status on cortical thickness (Gutiérrez-Galve et al., 2009); the correlation between rate of atrophy and change in certain neuropsychological test scores (Schott et al., 2008); and the demonstration that the use of affine image registration (nine degrees of freedom) can correct for longitudinal changes in voxel size (Whitwell et al., 2004). These data have been used to compare methods for automated calculation of volumes and atrophy in the hippocampus (Barnes et al., 2008a), and atrophy measured using the boundary shift integral with both Jacobian integration (Boyes et al., 2006), and with both SIENA and SIENAX (Smith et al., 2007). For clinical trials, the MIRIAD dataset has been used to estimate required sample sizes for six-month AD clinical trials using ventricular expansion (Schott et al., 2005) and hippocampal atrophy (Barnes et al., 2008a) as outcome measures; and using grey matter atrophy (Anderson et al., 2012). Using a multi-level model specifically designed to analyse this data-set (Frost et al., 2004; Schott et al., 2006) provided a detailed assessment of the mean whole brain rate of atrophy (2.23%/year, 95% CI: 1.90–2.56%/year using the boundary shift integral implementation at that time) and between- and within-subject sources of variance in atrophy in AD trials (total variance 0.99^2^ + (0.82/*t*)^2^, from 2 scans ‘*t*’ years apart). Using these results they estimated sample sizes required to power AD studies over intervals of 6, 12, and 24 months (with and without drop-outs) using between 2 and 5 scanning time points spaced either evenly or optimally. This model was subsequently extended to estimate whether estimating brain atrophy from a run-in period could increase efficiency for clinical trials (Frost et al., 2008).

## Data release

For the purposes of this data-release scans were converted from DICOM to NIfTI-1 format using MRICron/dcm2nii^1^ and all identifiable information removed in the process. Subjects were assigned a random identifier and the NIfTI files with a subject and visit identifier were placed in an Extensible Neuroimaging Archive Toolkit (XNAT, Marcus et al., 2007) database. An archive containing all the images was also prepared and placed on the XNAT system. For those visits where MMSE scores were collected this information is also available. The full dataset and terms of use will be available at http://miriad.drc.ion.ucl.ac.uk/ from Friday 8th February 2013.

## Discussion

We hope that the release of this dataset will provide the imaging community—and particularly those interested in the accurate quantification of volume change from serially acquired MR—with a useful common resource with which techniques can be developed, validated and compared, prior to implementation on other datasets or for clinical trials. Towards this goal this dataset has already been used in a blinded form as part of the Medical Image Computing and Computer Assisted Intervention (MICCAI) 2012 challenge “Atrophy Measurement Biomarkers using Structural MRI for Alzheimer's Disease”.^2^ The release of this dataset in an open form (together with the blinding codes from the MICCAI challenge) will enable other workers to compare their methods to workshop outcomes on the same dataset.

## Figures and Tables

**Table 1:** Demographics of the dataset.

	Alzheimer's Disease (N = 46)	Controls (N = 23)
Age at study entry (years)	69.4 ± 7.1	69.7 ± 7.2
Men	41 (%)	52 (%)
Mean (SD) baseline MMSE	19.2 ± 4	29.4 ± 0.8

**Table 2:** Timing and attendance of visits.

Mean interval days from baseline (SD)	0	16 (5)	43 (6)	98 (8)	180 (7)
Subjects (patients) scanned	68 (45)	66 (44)	67 (45)	68 (46)	66 (44)
Scans completed	133	66	130	68	66
					
Mean interval days from baseline (SD)	270 (19)	365 (14)	552 (18)	730 (10)	
Subjects (patients) scanned	60 (38)	67 (44)	39 (26)	22 (14)	
Scans completed	117	67	39	22	

**Table 3:** Publications featuring MIRIAD data.

*Atrophy in dementia*	
Boyes et al. (2006)	NeuroImage
Gutiérrez-Galve et al. (2009)	Dement Geriatr Cogn
Ridgway et al. (2009)	NeuroImage
Cardoso et al. (2009)	MICCAI 2009
Anderson et al. (2012)	Neurobiol Aging

*Medial–temporal lobe atrophy*	
Barnes et al. (2005)	Dement Geriatr Cogn
Barnes et al. (2007a)	Neurobiol Aging
Barnes et al. (2007b)	J Comput Assist Tomo
Ridha et al. (2007)	Arch Neurol-Chicago
Barnes et al. (2008a)	NeuroImage
Barnes et al. (2008b)	Neurobiology
Barnes et al. (2010)	NeuroImage
Leung et al. (2010)	NeuroImage

*Simulation studies*	
Schweiger et al. (2005)	MICCAI 2005
Camara-Rey et al. (2006a)	MICCAI 2006
Camara et al. (2006)	IEEE T Med Imaging
Camara-Rey et al. (2006b)	MICCAI 2006
Camara et al. (2007)	MICCAI 2007
Camara et al. (2008)	NeuroImage

*Clinical trials design*	
Whitwell et al. (2004)	Magn Reson Imaging
Schott et al. (2005)	Neurology
Schott et al. (2006)	J Neurol
Frost et al. (2008)	Stat Med
Schott et al. (2008)	Neuropsychologia

*Methods comparison*	
Smith et al. (2007)	NeuroImage
Clarkson et al. (2011)	NeuroImage

## Footnotes

1 http://www.nitrc.org/projects/mricron.

2 http://picsl.upenn.edu/Miccai2012/Challenge.
